# Supplementary material for: Design, Synthesis and Biological Evaluation of New Carbohydrate-Based Coumarin Derivatives as Selective Carbonic Anhydrase IX Inhibitors via “Click” Reaction
Source: Molecules. 2022 Aug 25;27(17):5464. doi: 10.3390/molecules27175464 (PMC9458059; doi:10.3390/molecules27175464)

Design, synthesis and biological evaluation of new carbohydrate-based coumarin derivatives as selective carbonic anhydrase IX inhibitors via “click” reaction

Naying Chu [1,2,†], Yitong Wang [3,†], Hao Jia [1], Jie Han [1], Xiaoyi Wang [1] and Zhuang Hou [3,*]

[1] *Department of Pharmacy, The First People’s Hospital of Shangqiu, Suiyang District, 292 Kaixuan Road, Shangqiu 476000, China;*

[2] *China-Japan Research Institute of Medical and Pharmaceutical Sciences, Shenyang Pharmaceutical University, 103 Wenhua Road, Shenyang 110016, China*

[3] *Key Laboratory of Structure-Based Drugs Design and Discovery (Ministry of Education), Shenyang Pharmaceutical University, Shenyang 110016, China*

** Correspondence: houzhuang8@sina.com*

† These authors contributed equally to this work.

The ^1H and ^{13}C NMR spectra were recorded at 600 MHz and 150 MHz on a Bruker ARX 600 MHz spectrometer using $(CD_3)_2SO$ as solvents with TMS as the internal standard. The NMR spectra were analyzed and interpreted using MestReNova. The solvent peaks were 2.5 ppm and 40 ppm in the ^1H and ^{13}C NMR spectra, respectively.

*Figures S1. The ¹H-NMR spectrum of 7- [(1-β-D-glucopyranosyl-1H-1,2,3-triazol-4-yl) methoxy]-2H-chromen-2-one (**10a**)*

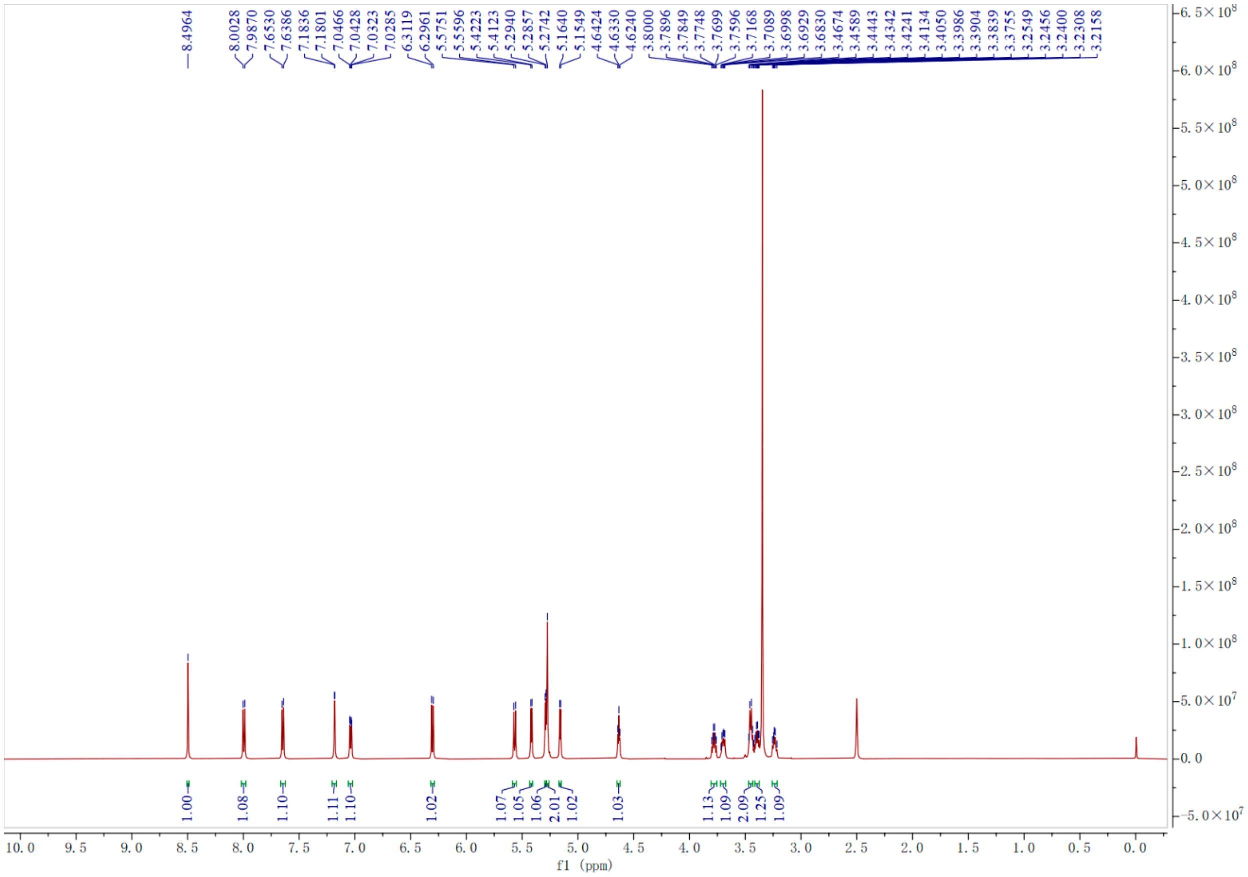

*Figures S2. The ¹³C-NMR spectrum of 7- [(1-β-D-glucopyranosyl-1H-1,2,3-triazol-4-yl) methoxy]-2H-chromen-2-one (**10a**)*

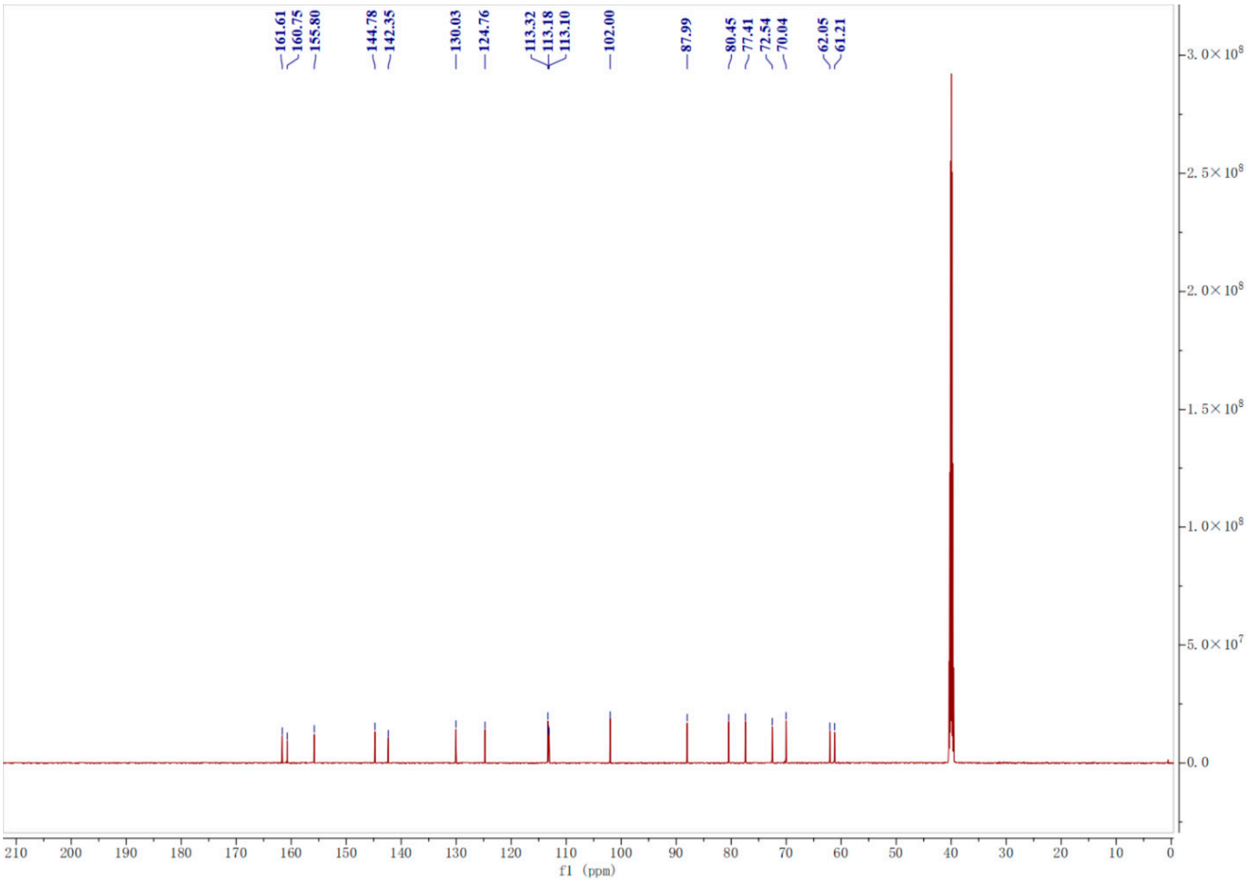

*Figures S3. The ^{1}H-NMR spectrum of 7- [(1-β-D-galactopyranosyl-1H-1,2,3-triazol-4-yl) methoxy]-2H-chromen-2-one (**10b**)*

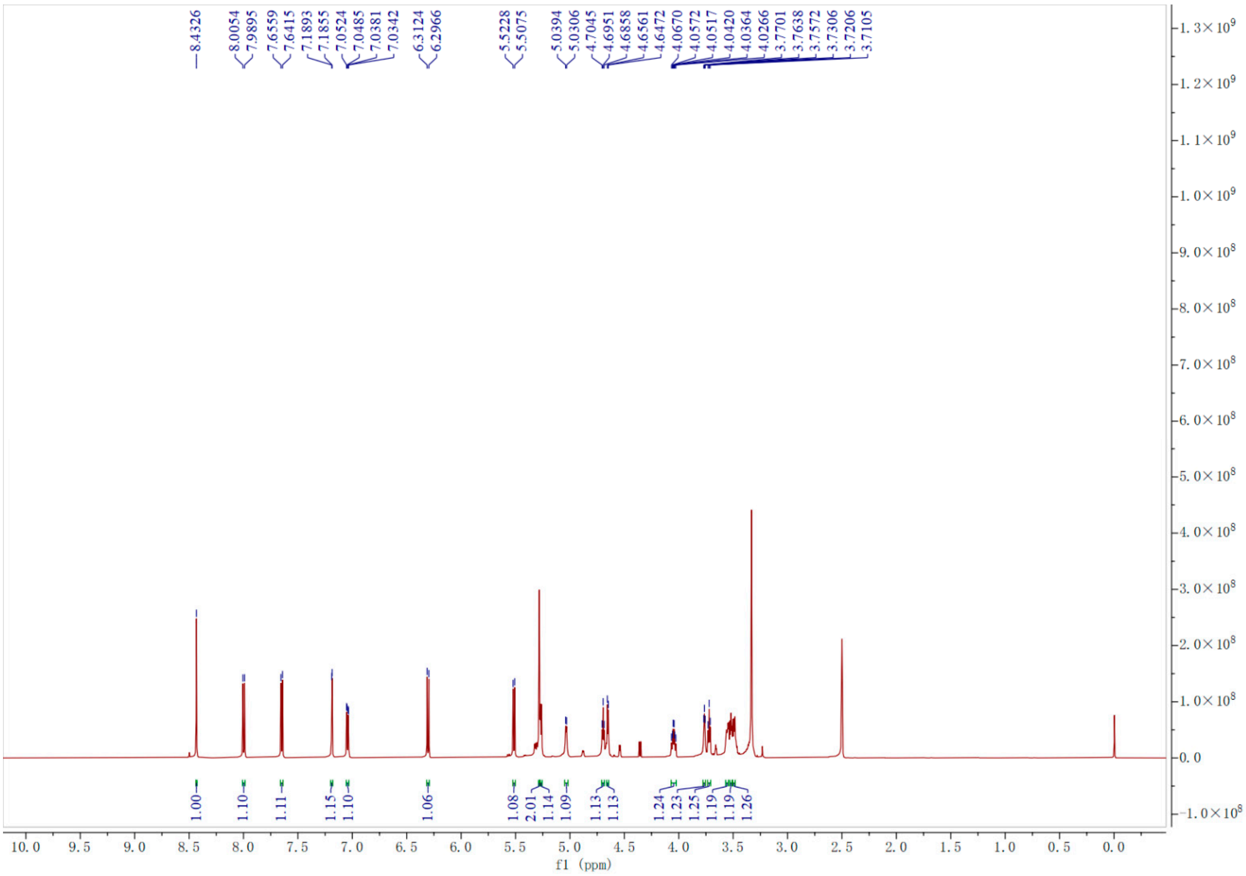

*Figures S4. The ^{13}C-NMR spectrum of 7- [(1-β-D-galactopyranosyl-1H-1,2,3-triazol-4-yl) methoxy]-2H-chromen-2-one (**10b**)*

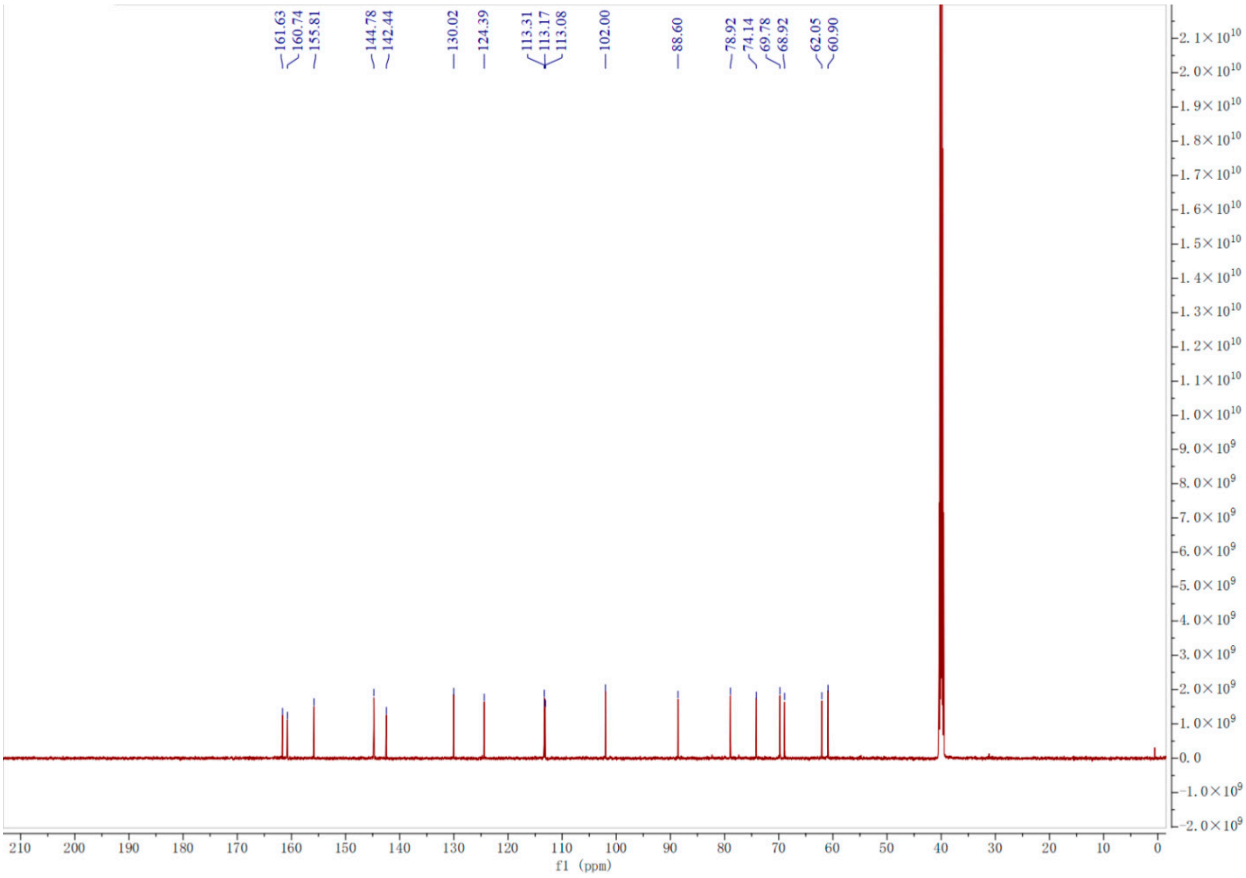

Figures S5. The ^{1}H-NMR spectrum of 7- [(1-β-D-mannopyranosyl-1H-1,2,3-triazol-4-yl) methoxy]-2H-chromen-2-one (**10c**)

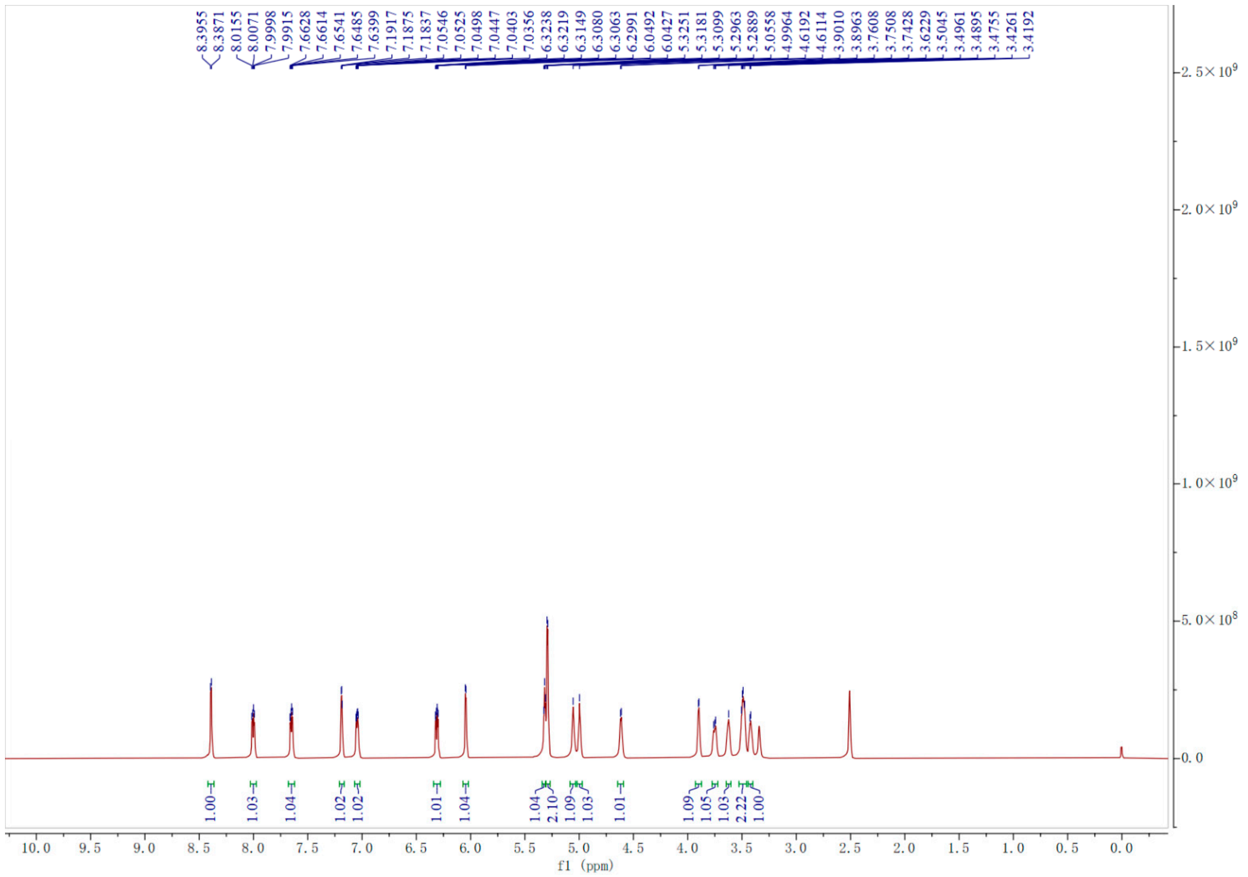

Figures S6. The ^{13}C-NMR spectrum of 7- [(1-β-D-mannopyranosyl-1H-1,2,3-triazol-4-yl) methoxy]-2H-chromen-2-one (**10c**)

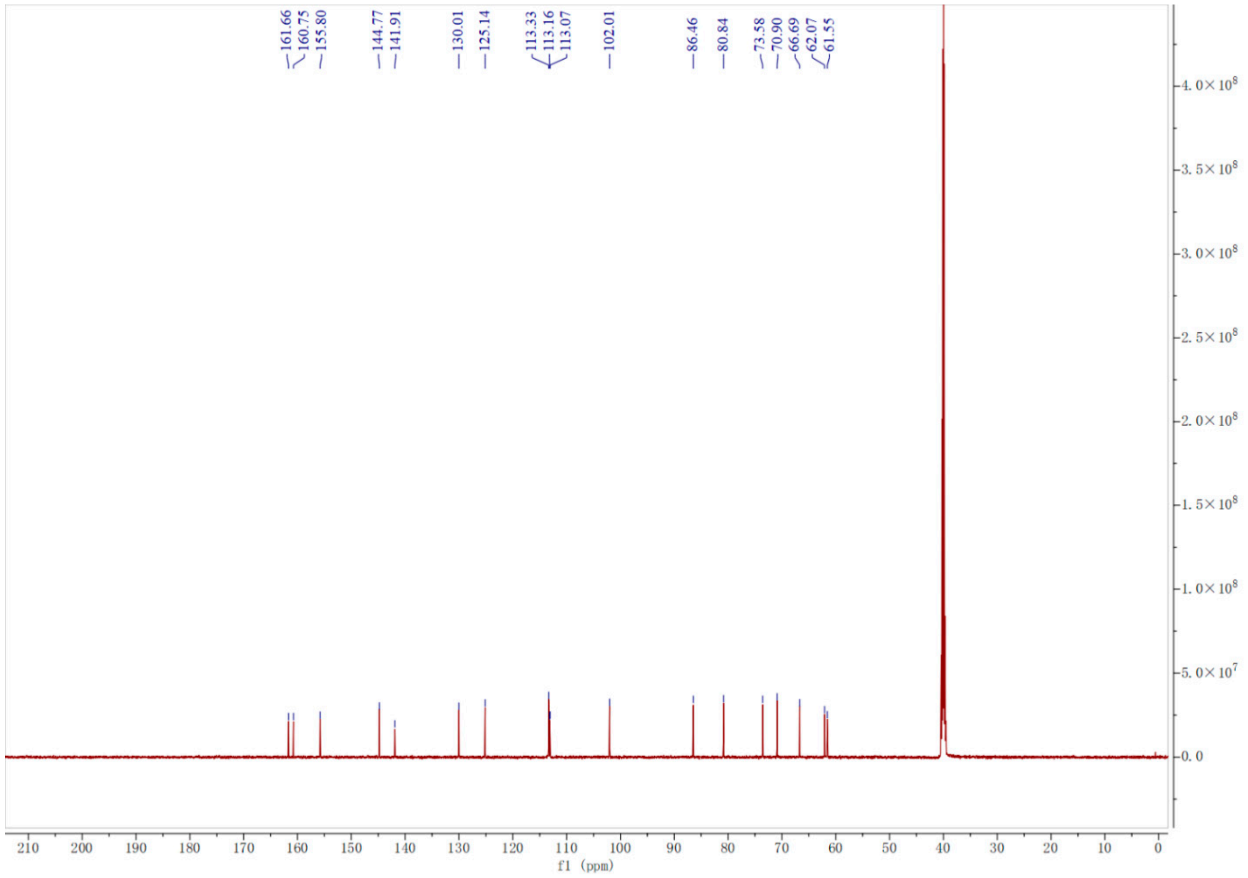

Figures S7. The ^{1}H-NMR spectrum of 7- [(1-β-D-glucosaminogly-1H-1,2,3-triazol-4-yl) methoxy]-2H-chromen-2-one (**10d**)

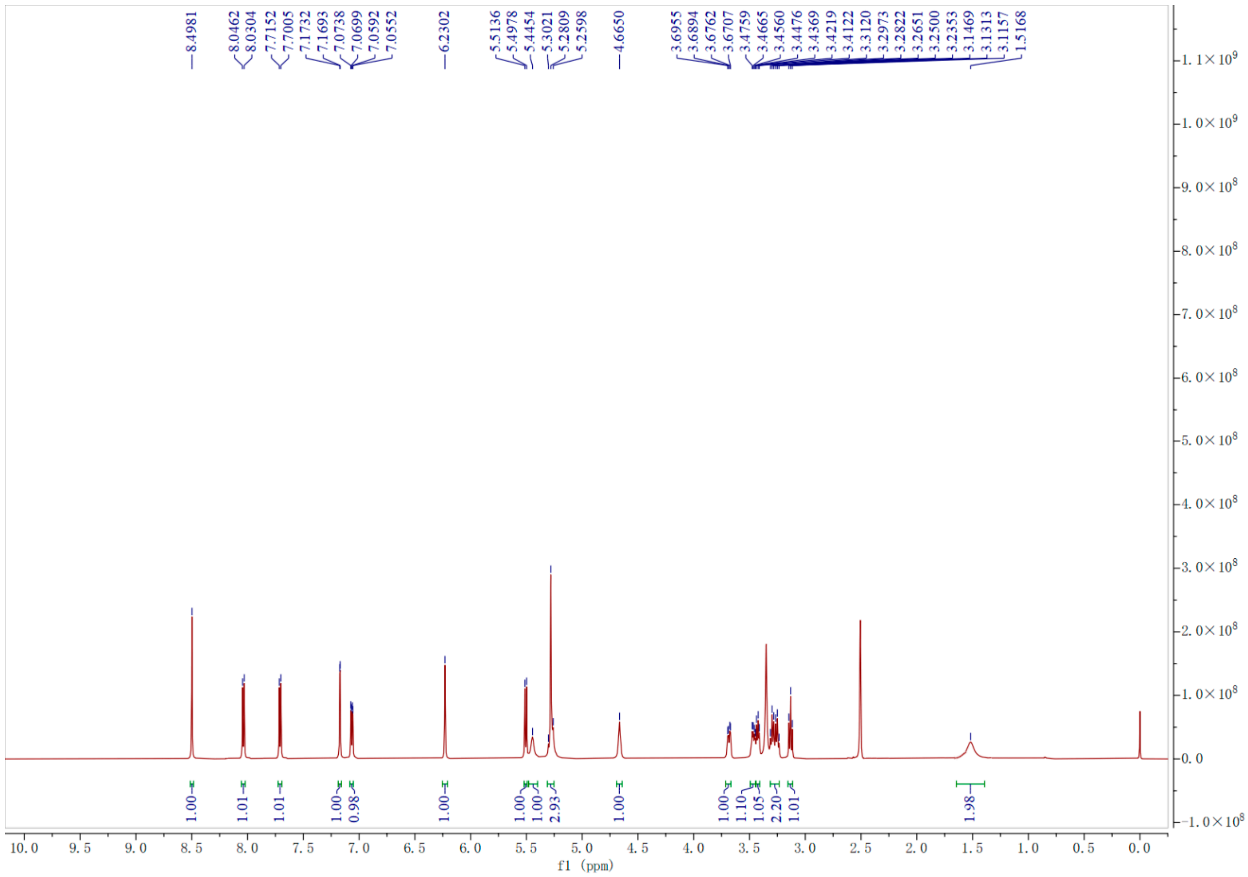

Figures S8. The ^{13}C -NMR spectrum of 7- [(1-β-D-glucosaminogly-1H-1,2,3-triazol-4-yl) methoxy]-2H-chromen-2-one (**10d**)

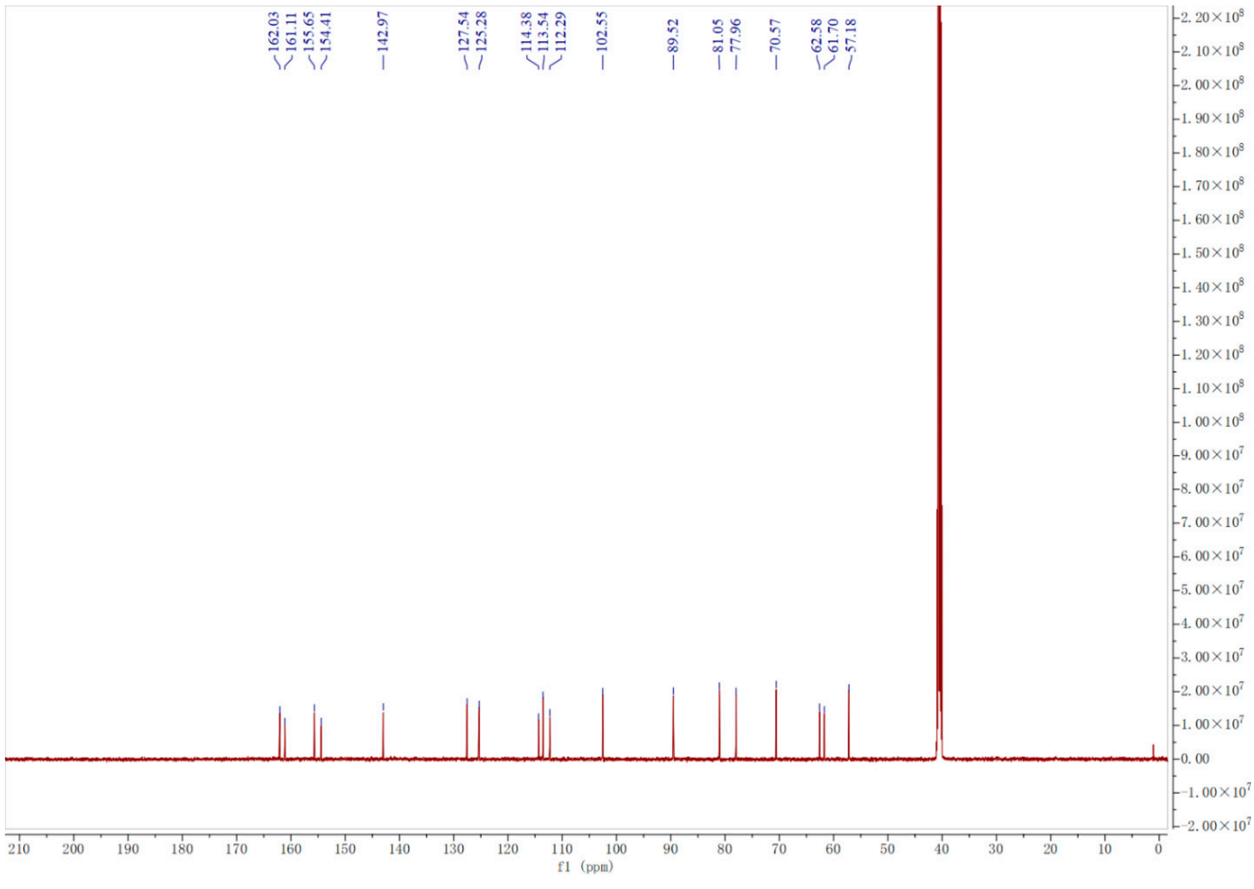

Figures S9. The ^{1}H-NMR spectrum of 7- [(1-β-D-xylopyranosyl-1H-1,2,3-triazol-4-yl) methoxy]-2H-chromen-2-one (**10e**)

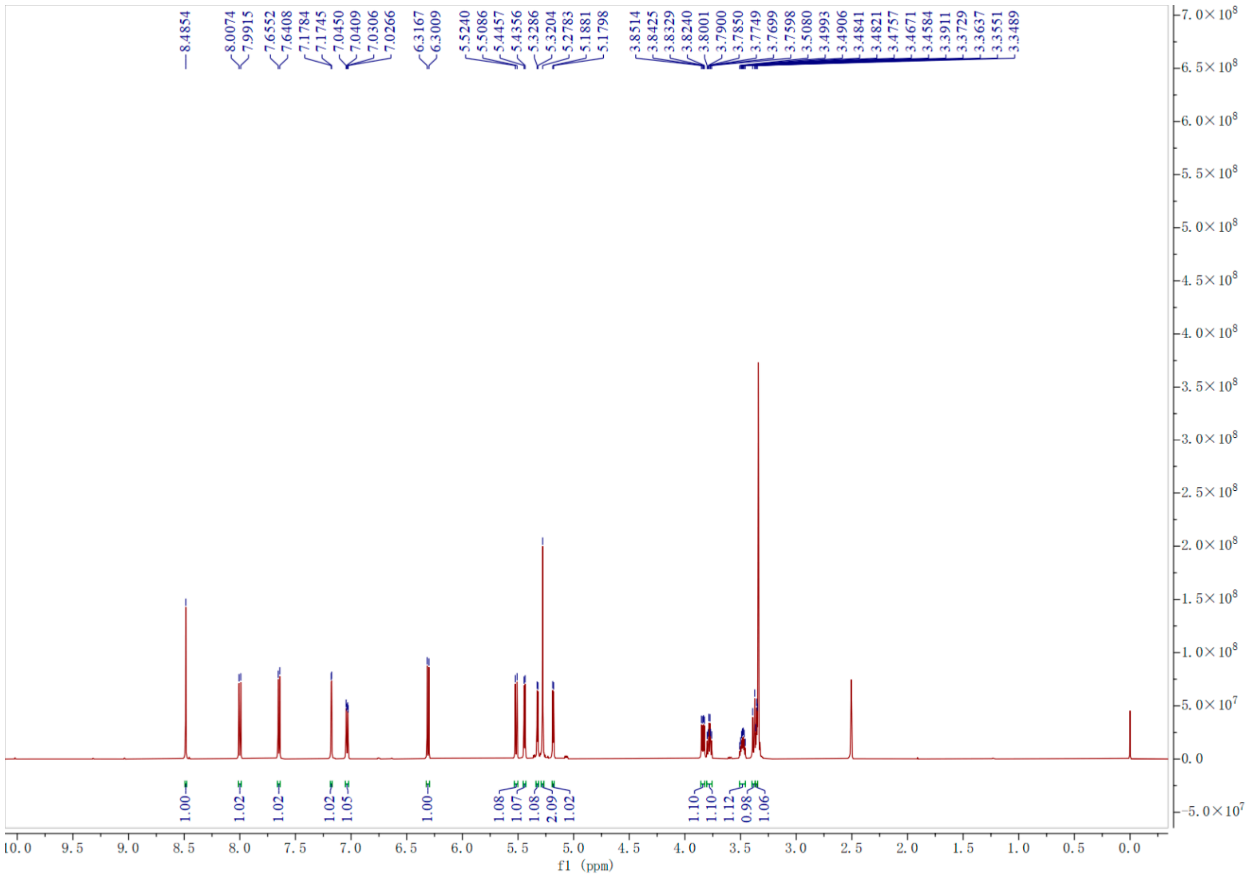

Figures S10. The ^{13}C-NMR spectrum of 7- [(1-β-D-xylopyranosyl-1H-1,2,3-triazol-4-yl) methoxy]-2H-chromen-2-one (**10e**)

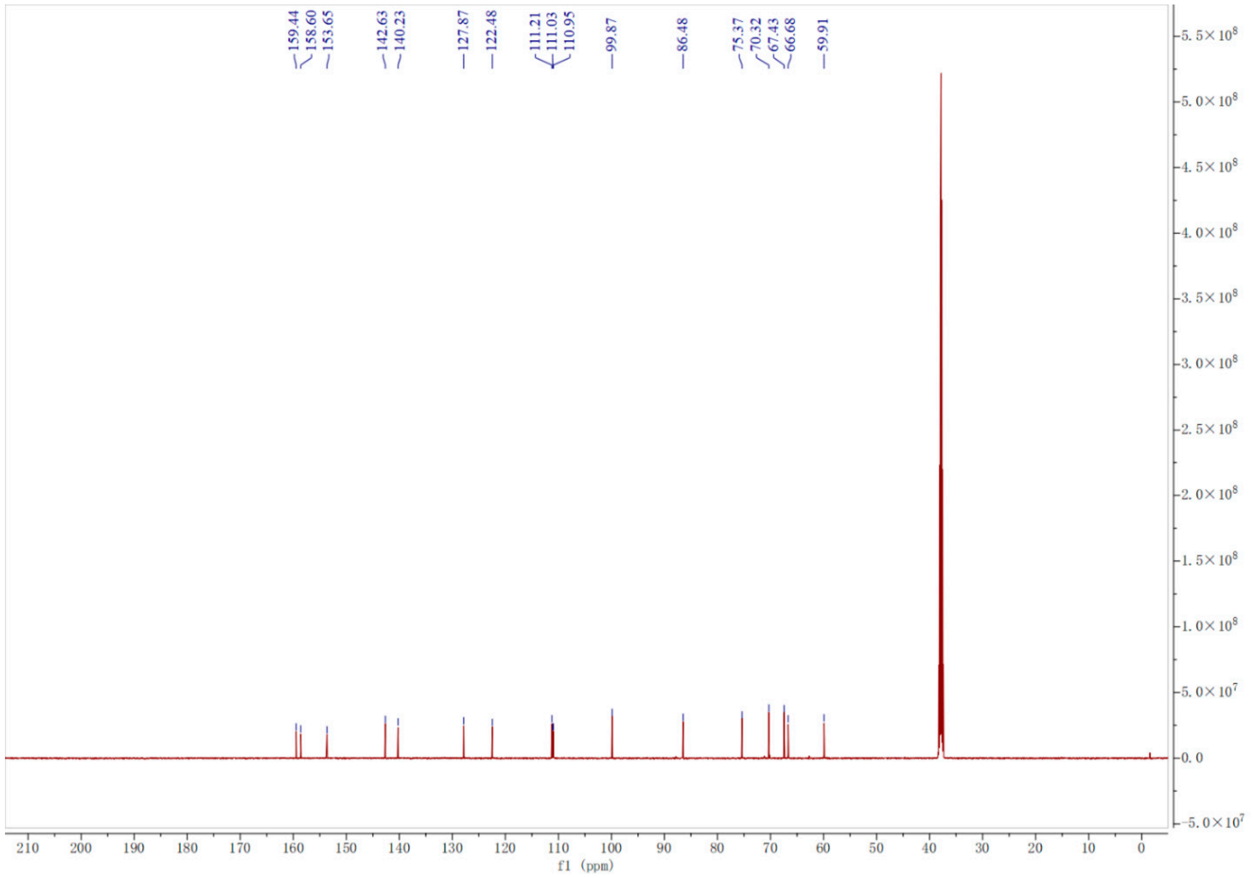

Figures S11. The ¹H-NMR spectrum of 7- [(1-β-L-arabinopyranosyl-1H-1,2,3-triazol-4-yl) methoxy]-2H-chromen-2-one (**10f**)

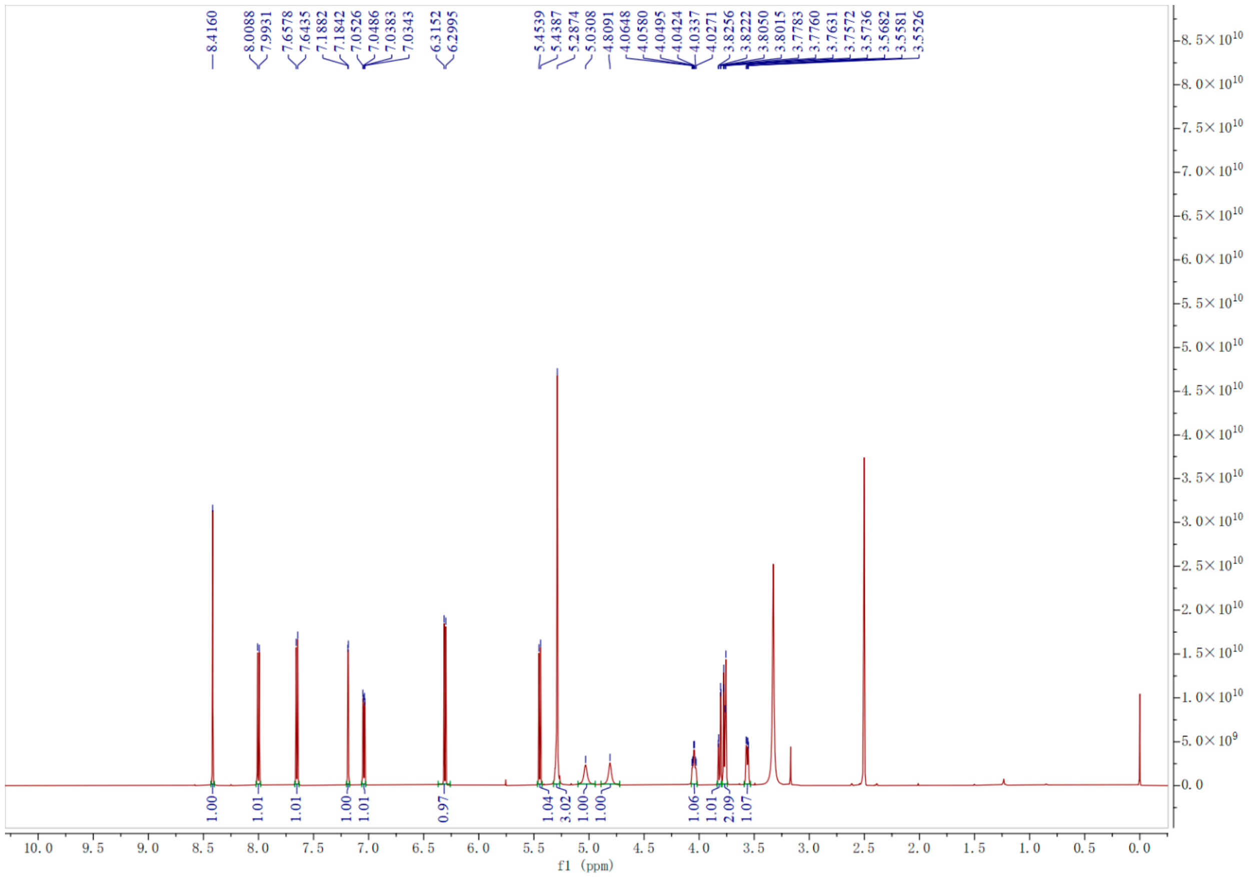

Figures S12. The ¹³C-NMR spectrum of 7- [(1-β-L-arabinopyranosyl-1H-1,2,3-triazol-4-yl) methoxy]-2H-chromen-2-one (**10f**)

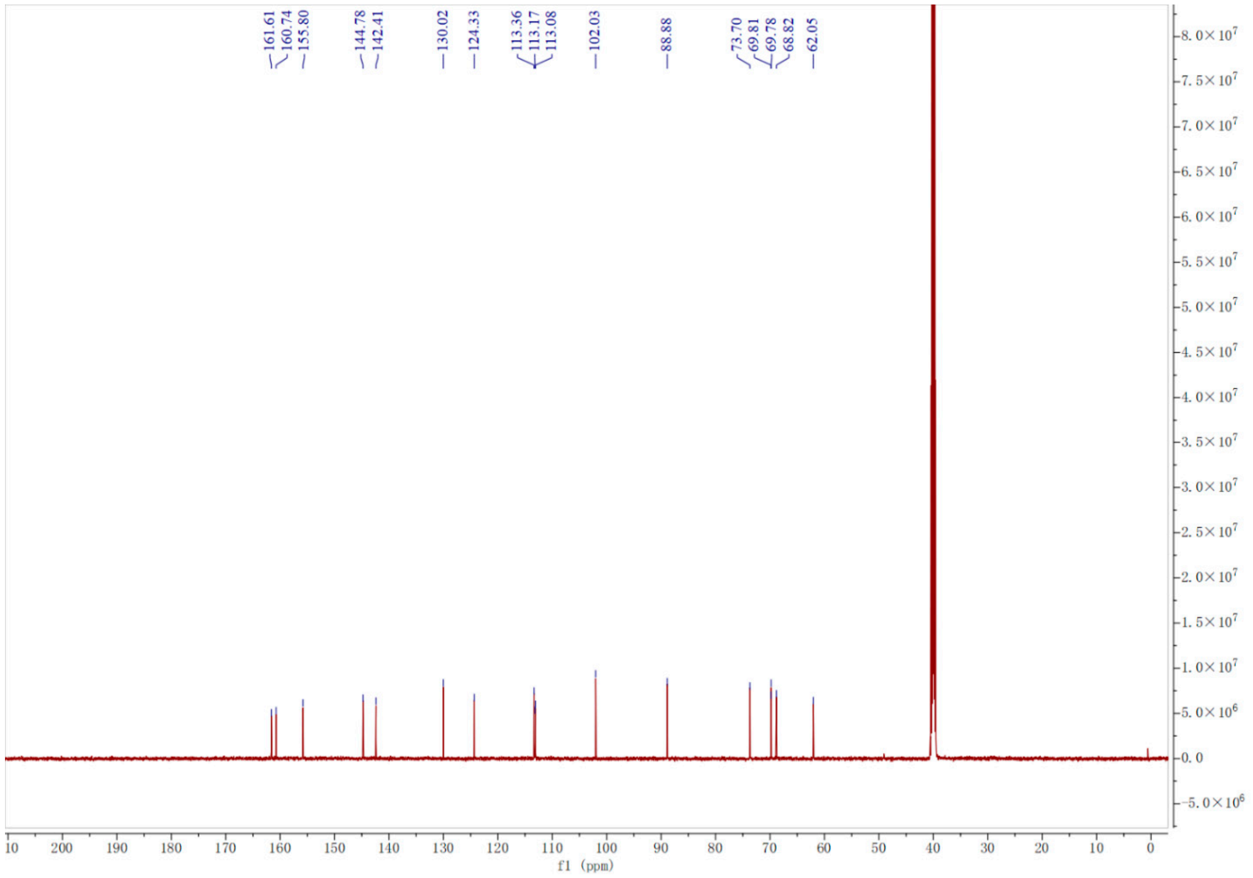

Figures S13. The ^{1}H-NMR spectrum of 7- [(1-β-L-rhamnosyl-1H-1,2,3-triazol-4-yl) methoxy]-2H-chromen-2-one (**10g**)

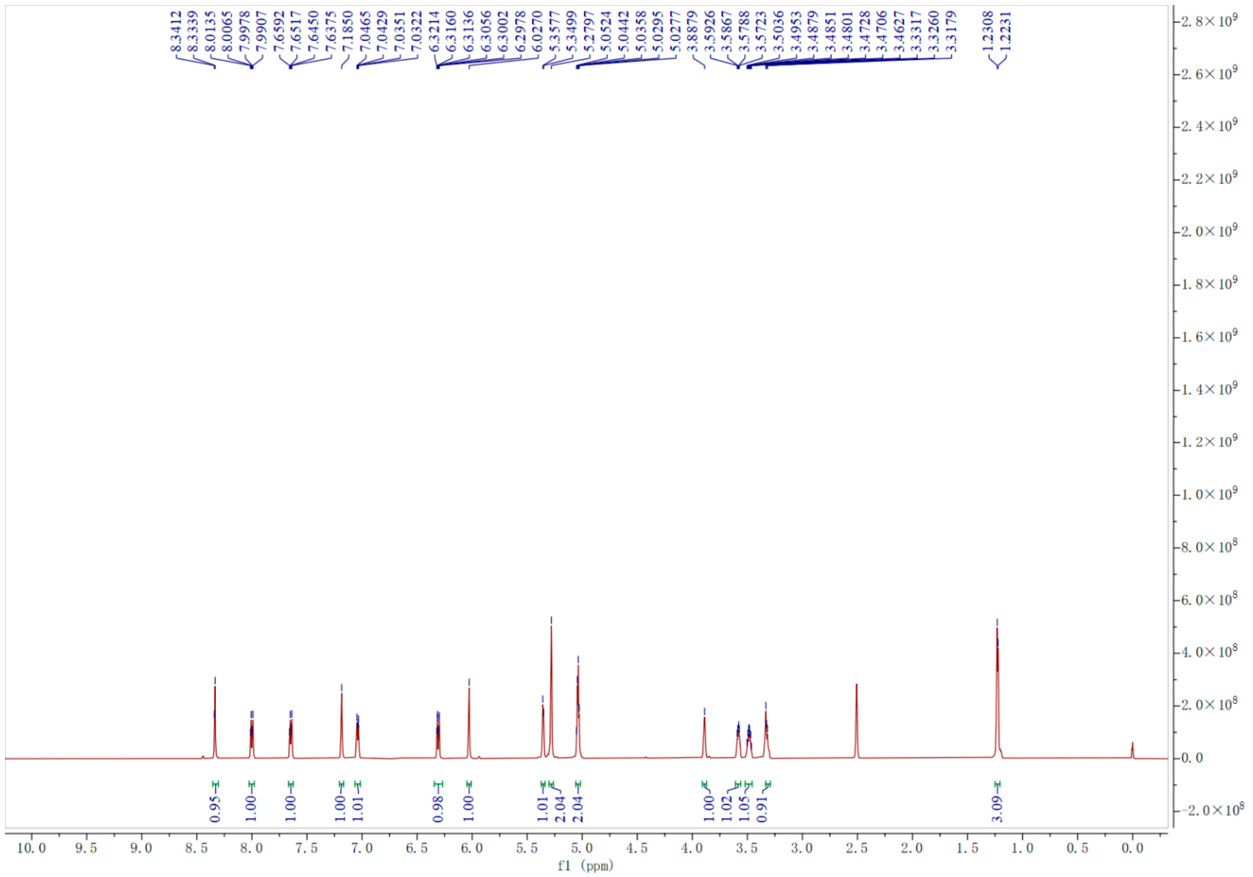

Figures S14. The ^{13}C-NMR spectrum of 7- [(1-β-L-rhamnosyl-1H-1,2,3-triazol-4-yl) methoxy]-2H-chromen-2-one (**10g**)

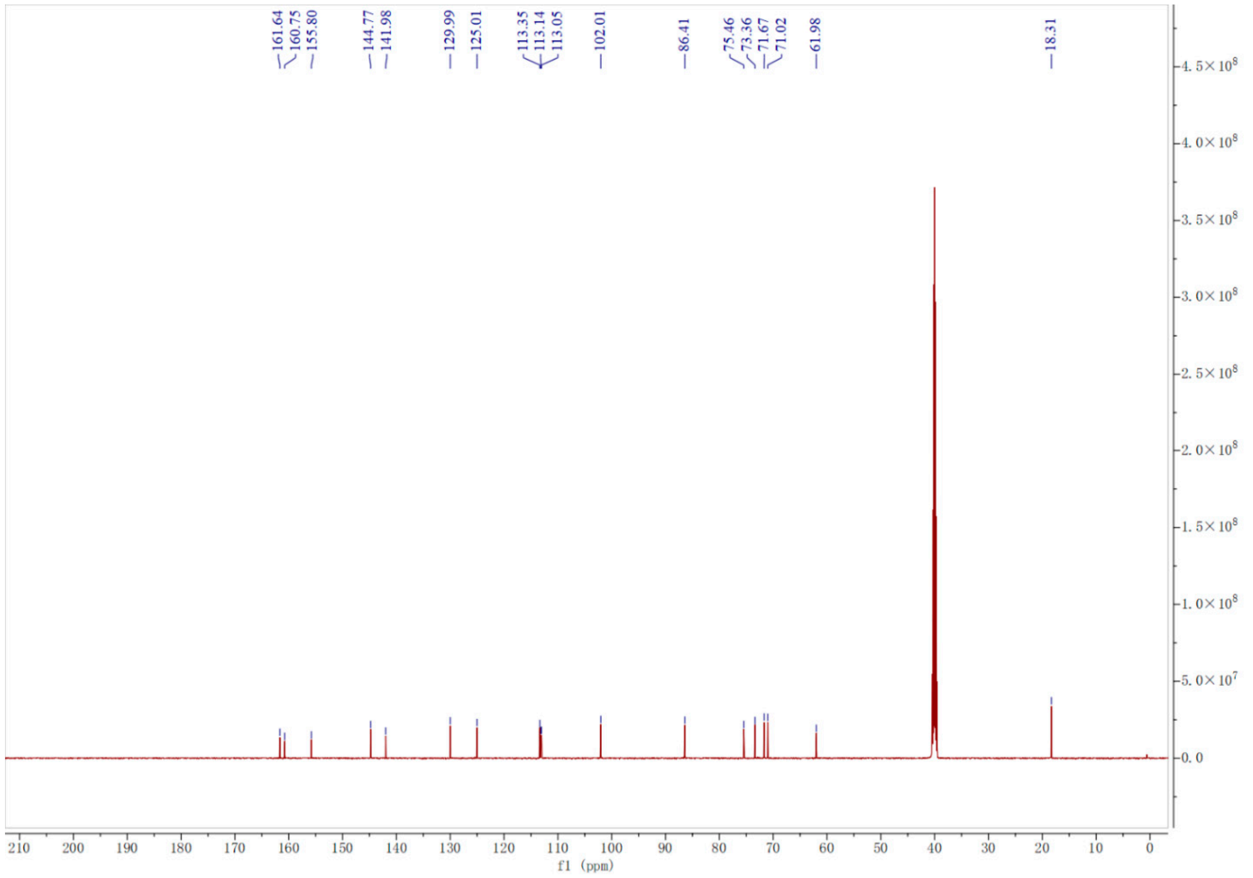

Figures S15. The ¹H-NMR spectrum of 7- [(1-β-D-ribofuranosyl-1H-1,2,3-triazol-4-yl) methoxy]-2H-chromen-2-one (**10h**)

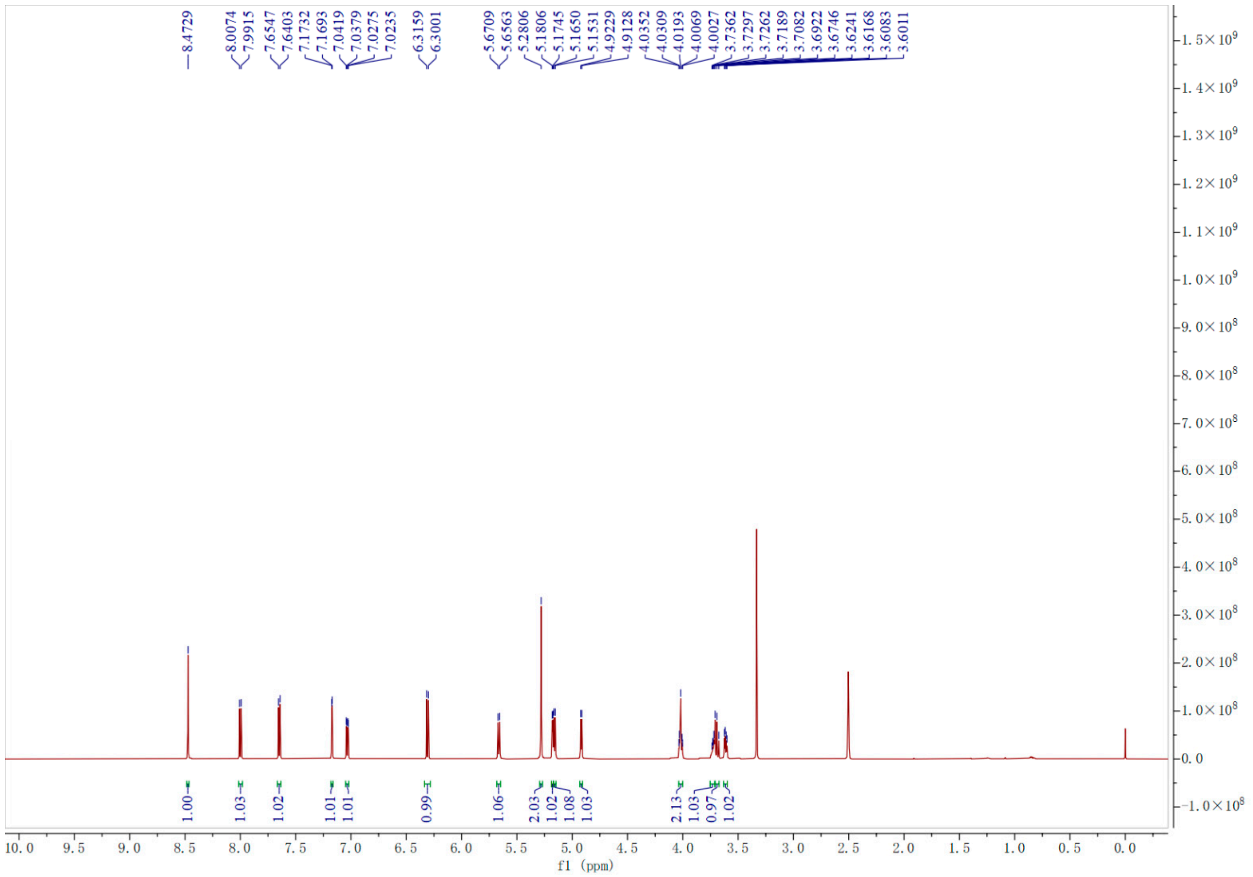

Figures S16. The ¹³C-NMR spectrum of 7- [(1-β-D-ribofuranosyl-1H-1,2,3-triazol-4-yl) methoxy]-2H-chromen-2-one (**10h**)

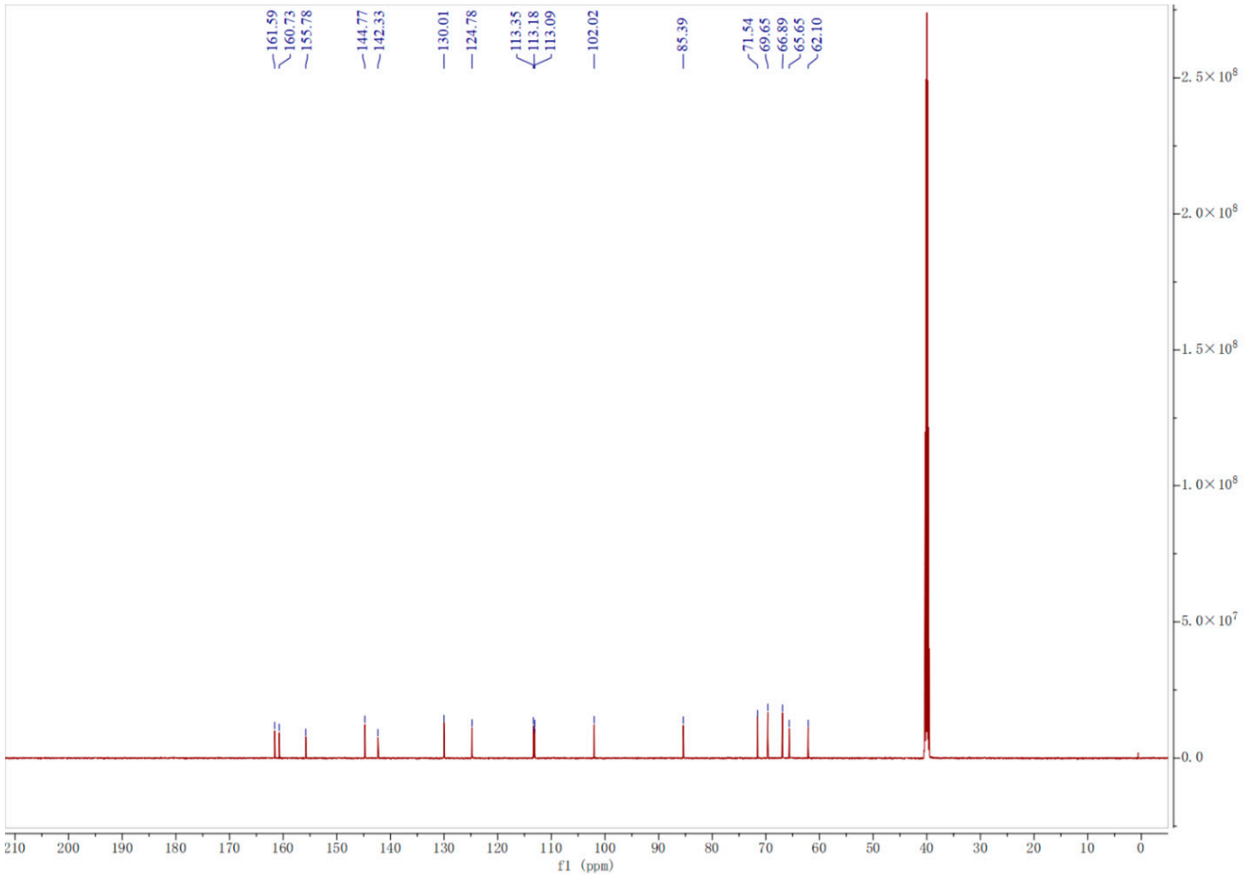

Figures S17. The ^{1}H-NMR spectrum of 7- [(1-β-D-glucopyranosyl-1H-1,2,3-triazol-4-yl) methoxy]-4-methyl-2H-chromen-2-one (**10i**)

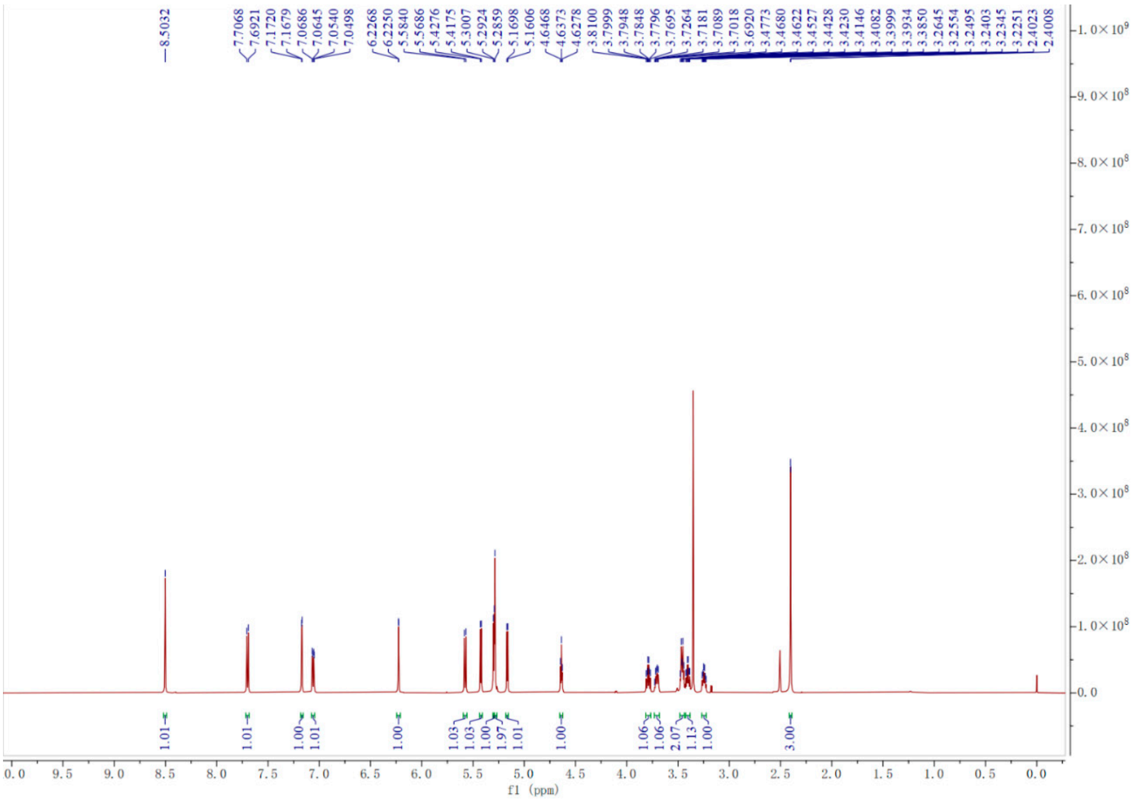

Figures S18. The ^{13}C-NMR spectrum of 7- [(1-β-D-glucopyranosyl-1H-1,2,3-triazol-4-yl) methoxy]-4-methyl-2H-chromen-2-one (**10i**)

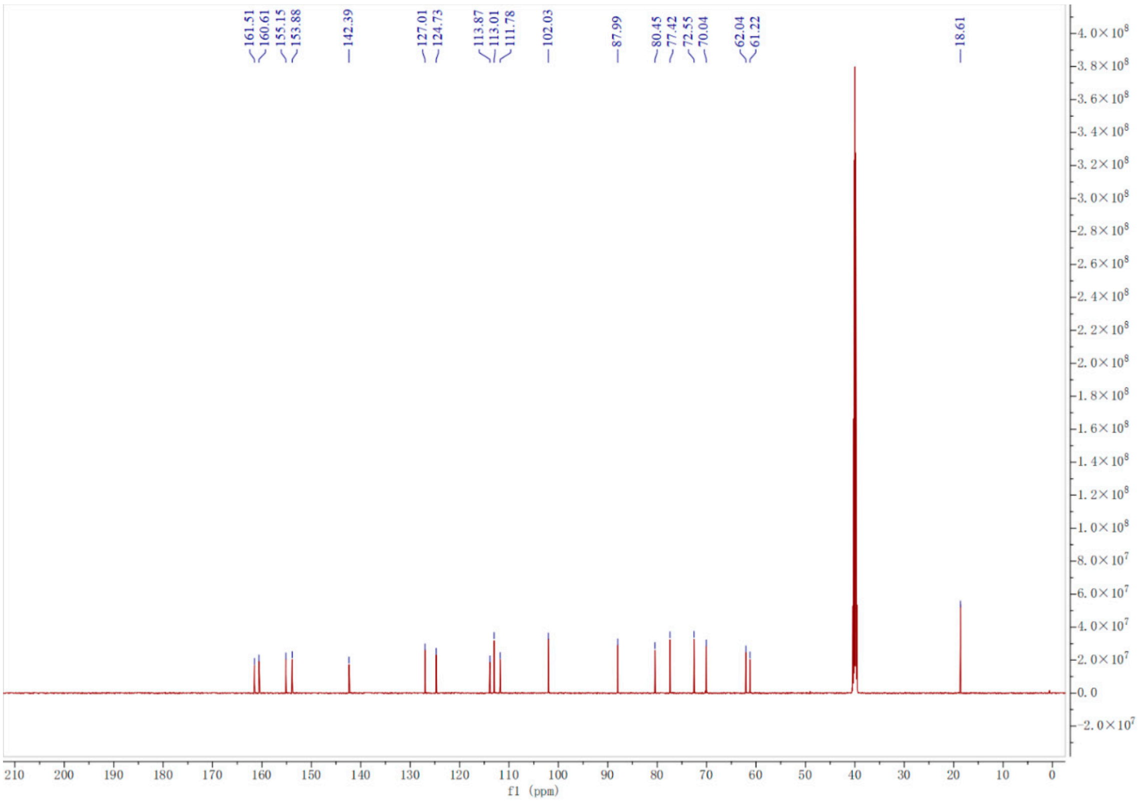

Figures S19. The ^{1}H-NMR spectrum of 7- [(1-β-D-galactopyranosyl-1H-1,2,3-triazol-4-yl) methoxy]-4-methyl-2H-chromen-2-one (**10j**)

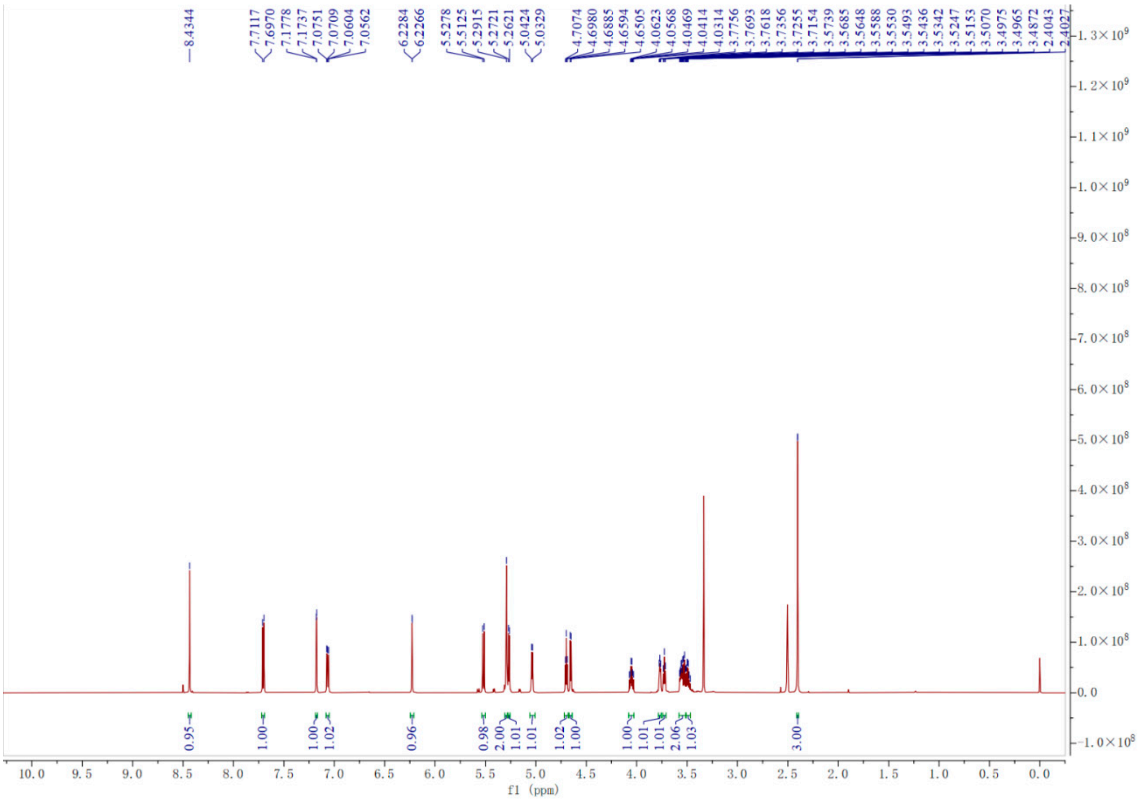

Figures S20. The ^{13}C-NMR spectrum of 7- [(1-β-D-galactopyranosyl-1H-1,2,3-triazol-4-yl) methoxy]-4-methyl-2H-chromen-2-one (**10j**)

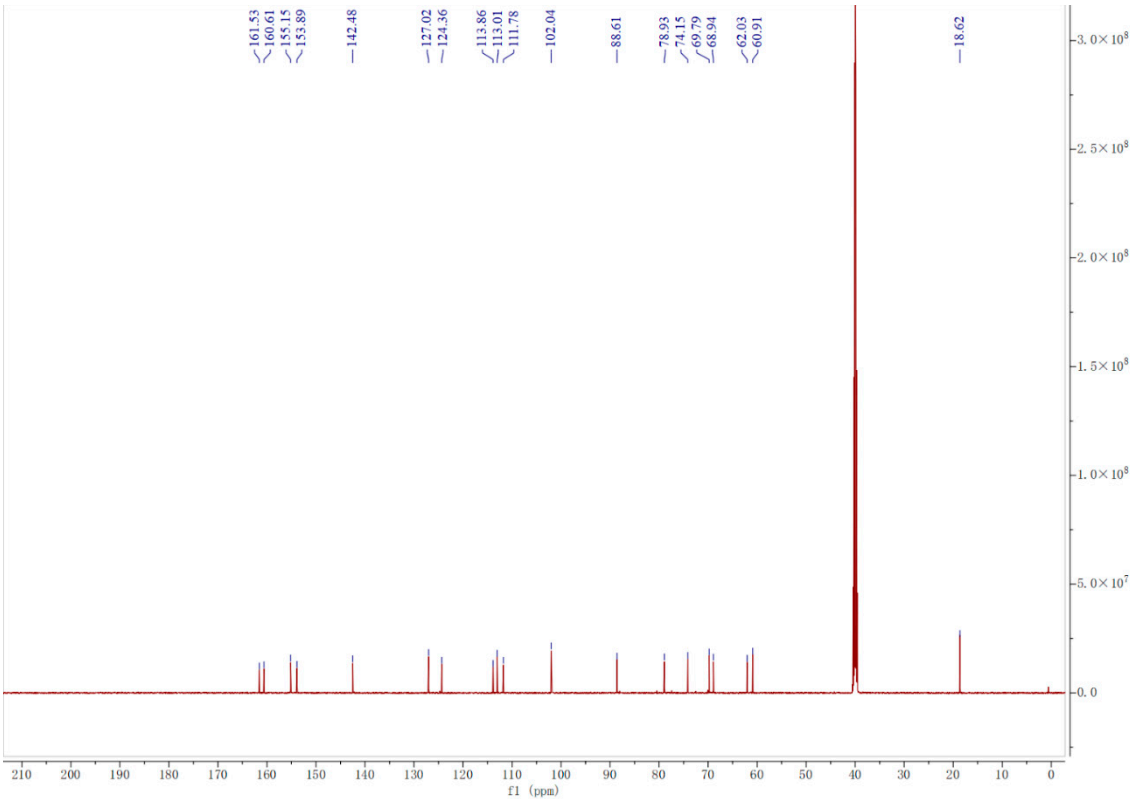

Figures S21. The ^{1}H-NMR spectrum of 7- [(1-β-D-mannopyranosyl-1H-1,2,3-triazol-4-yl) methoxy]-4-methyl-2H-chromen-2-one (**10k**)

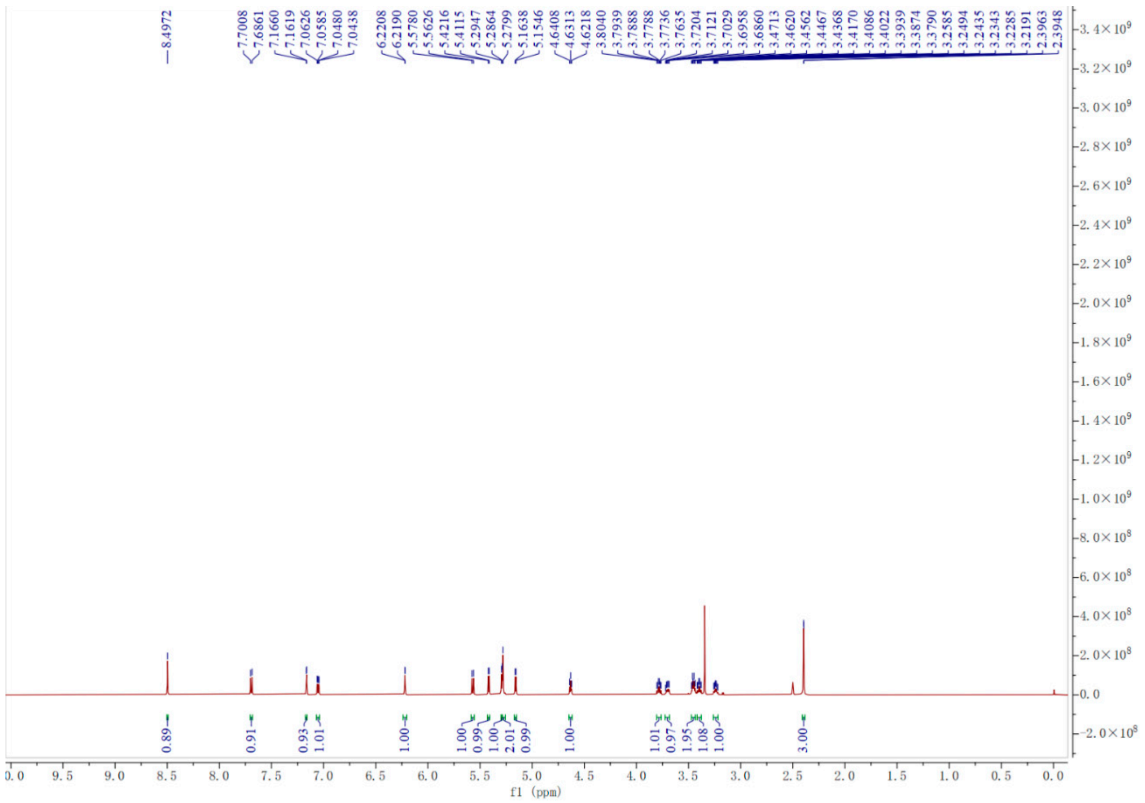

Figures S22. The ^{13}C-NMR spectrum of 7- [(1-β-D-mannopyranosyl-1H-1,2,3-triazol-4-yl) methoxy]-4-methyl-2H-chromen-2-one (**10k**)

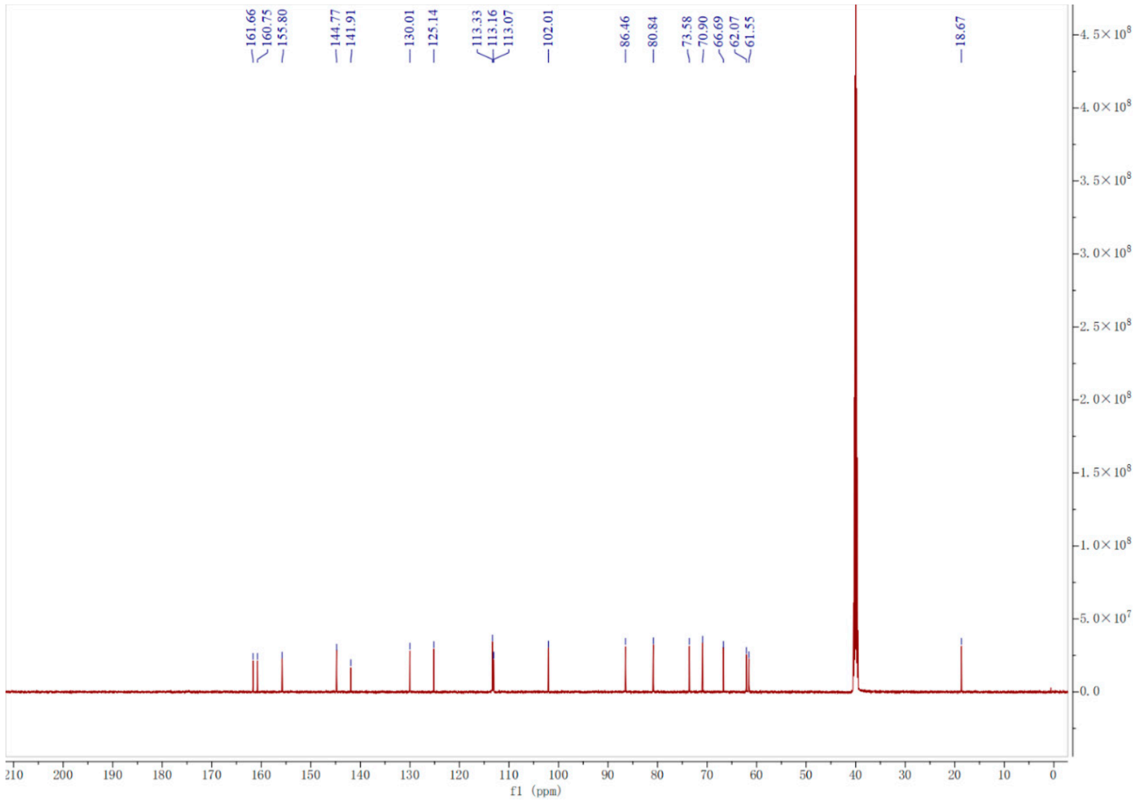

Figures S23. The ^{1}H-NMR spectrum of 7- [(1-β-D-glucosaminogly-1H-1,2,3-triazol-4-yl) methoxy]-4-methyl-2H-chromen-2-one (**10l**)

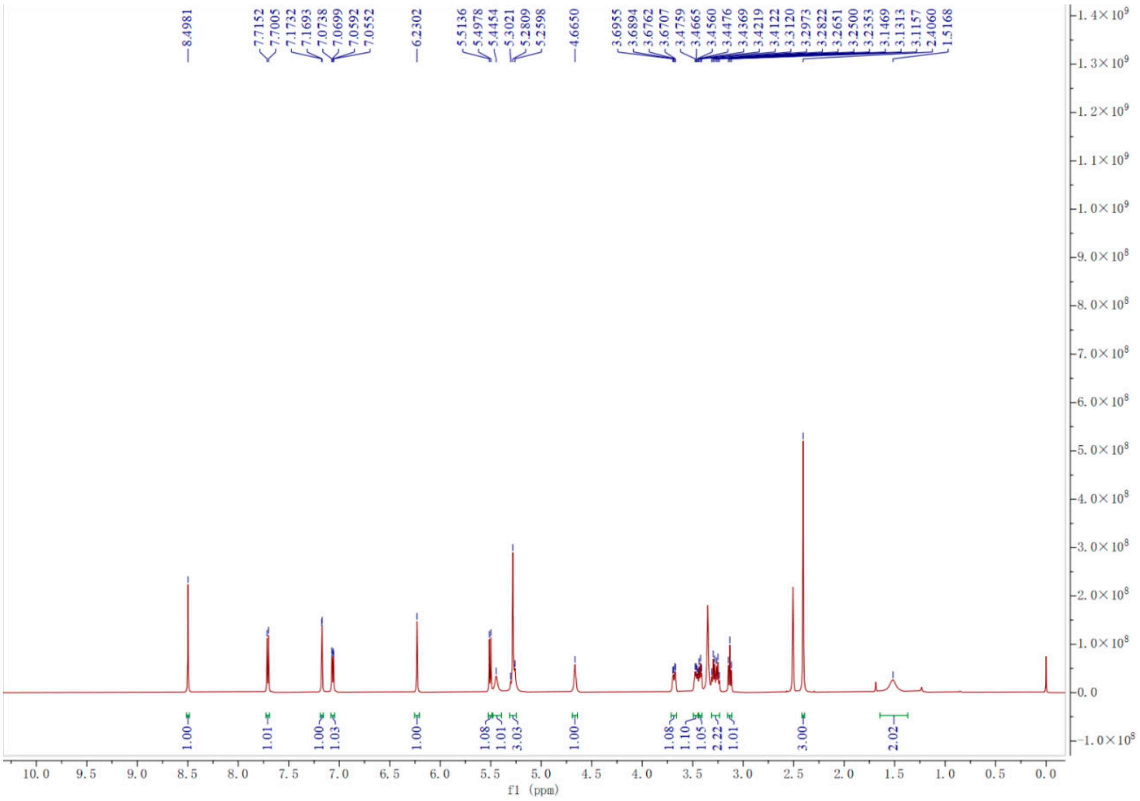

Figures S24. The ^{13}C-NMR spectrum of 7- [(1-β-D-glucosaminogly-1H-1,2,3-triazol-4-yl) methoxy]-4-methyl-2H-chromen-2-one (**10l**)

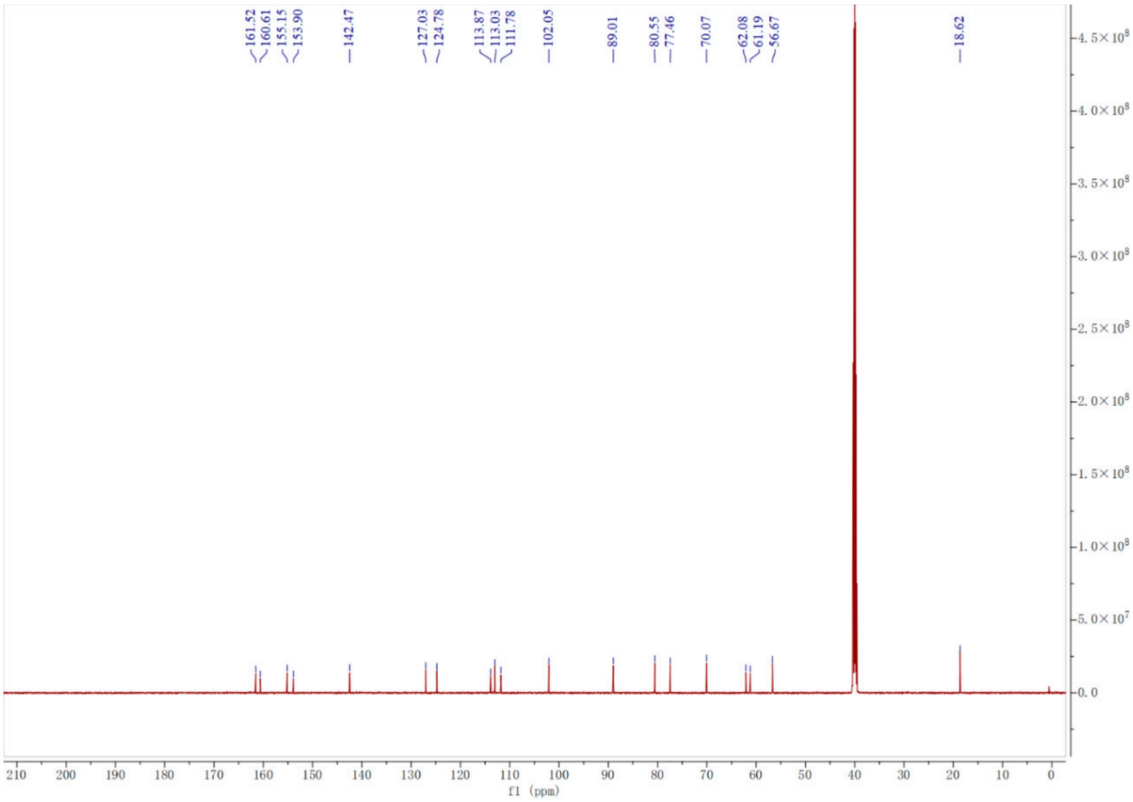

Figures S25. The ^{1}H-NMR spectrum of 7- [(1-β-D-xylopyranosyl-1H-1,2,3-triazol-4-yl) methoxy]-4-methyl-2H-chromen-2-one (**10m**)

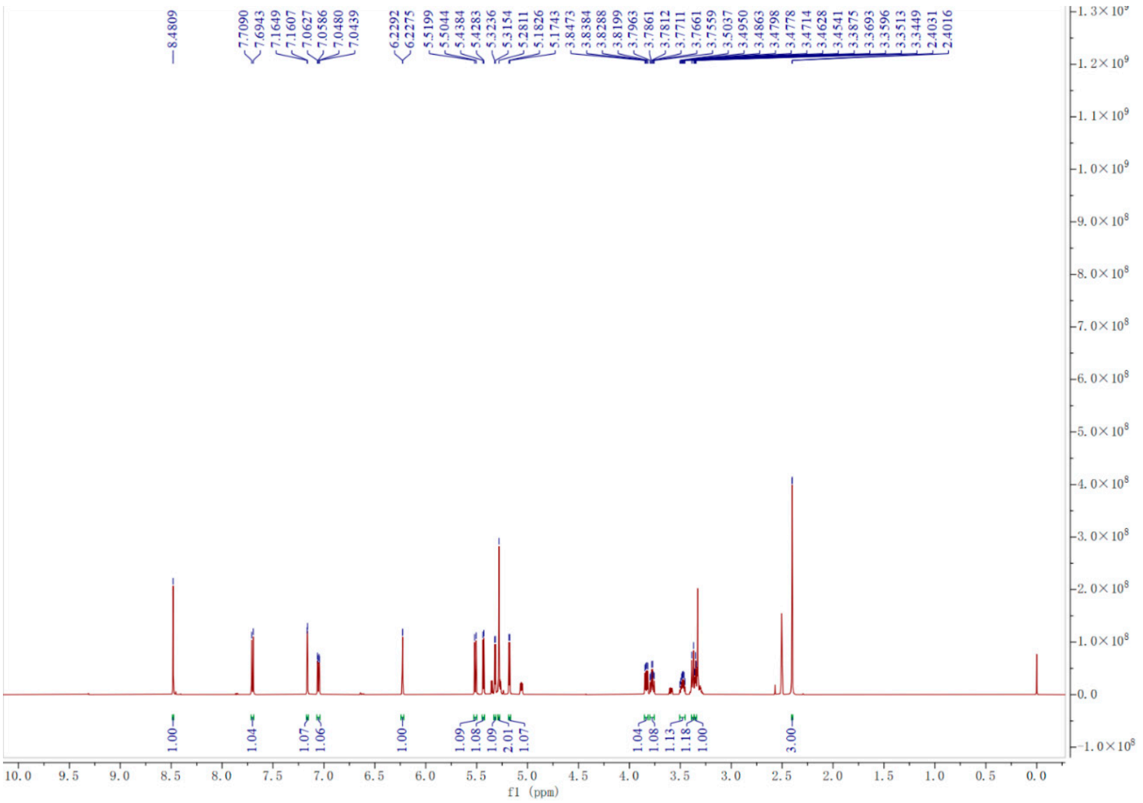

Figures S26. The ^{13}C-NMR spectrum of 7- [(1-β-D-xylopyranosyl-1H-1,2,3-triazol-4-yl) methoxy]-4-methyl-2H-chromen-2-one (**10m**)

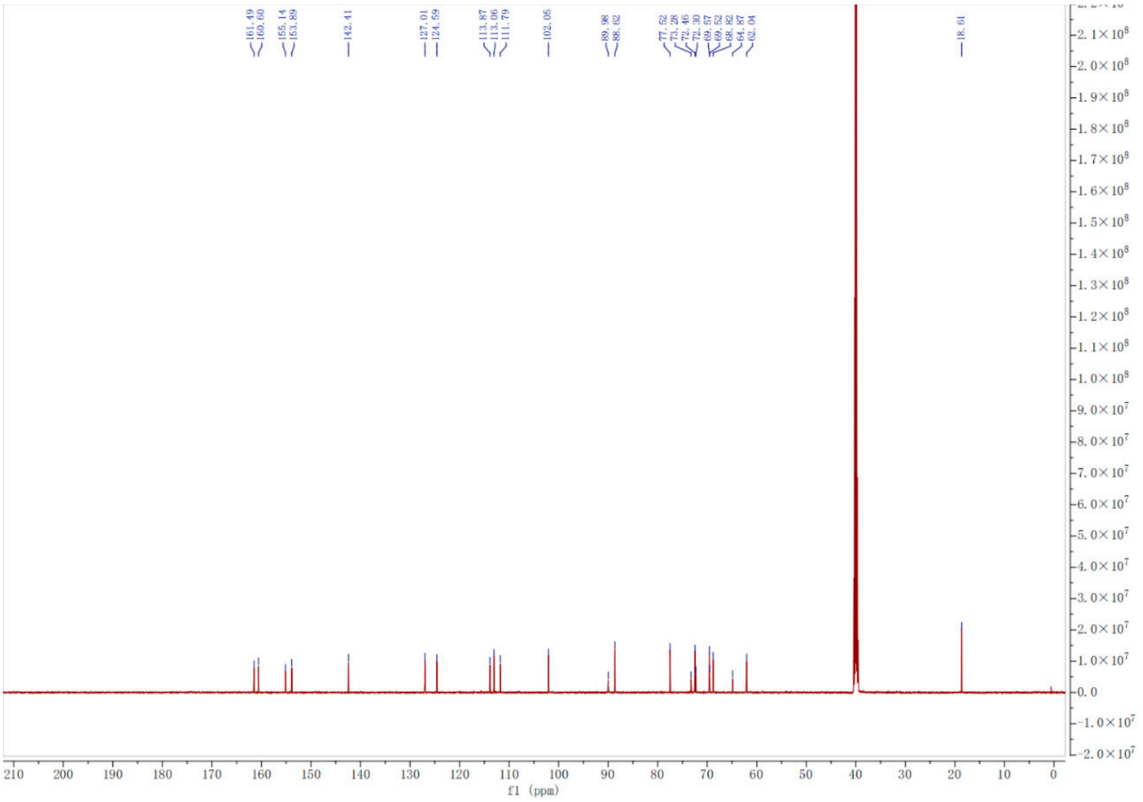

Figures S27. The ^{1}H-NMR spectrum of 7- [(1-β-L-arabinopyranosyl-1H-1,2,3-triazol-4-yl) methoxy]-4-methyl-2H-chromen-2-one (**10n**)

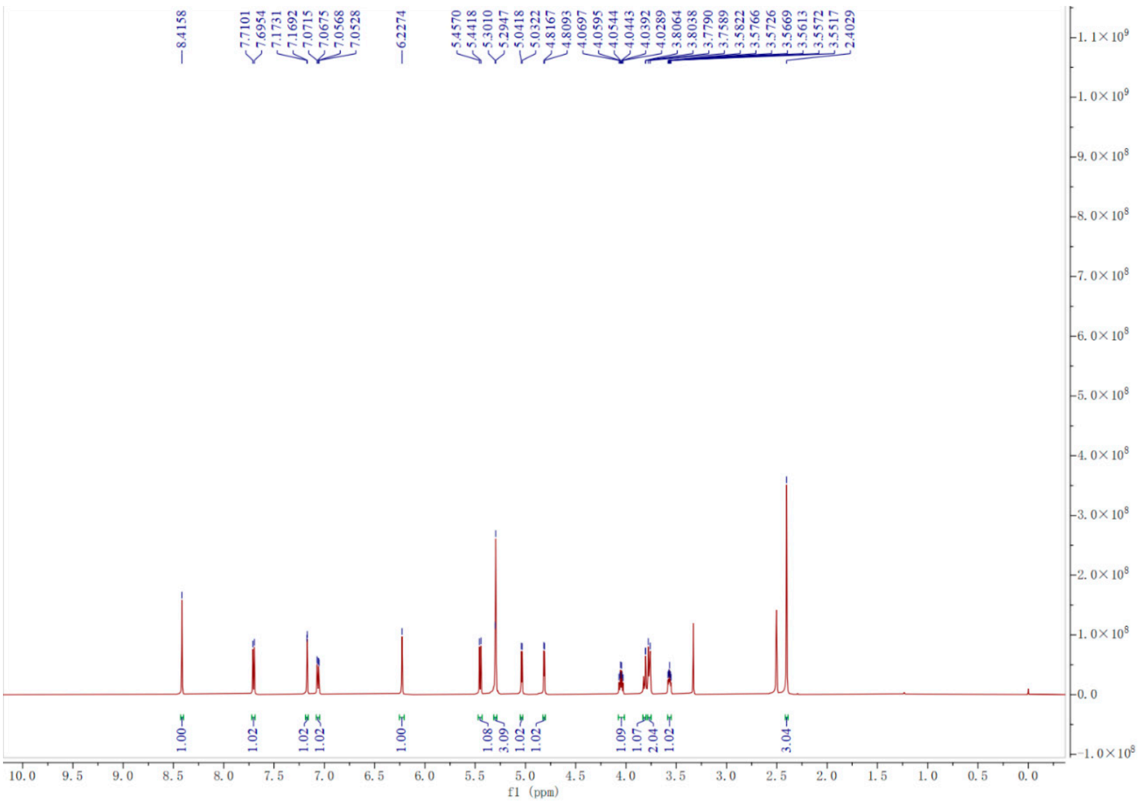

Figures S28. The ^{13}C-NMR spectrum of 7- [(1-β-L-arabinopyranosyl-1H-1,2,3-triazol-4-yl) methoxy]-4-methyl-2H-chromen-2-one (**10n**)

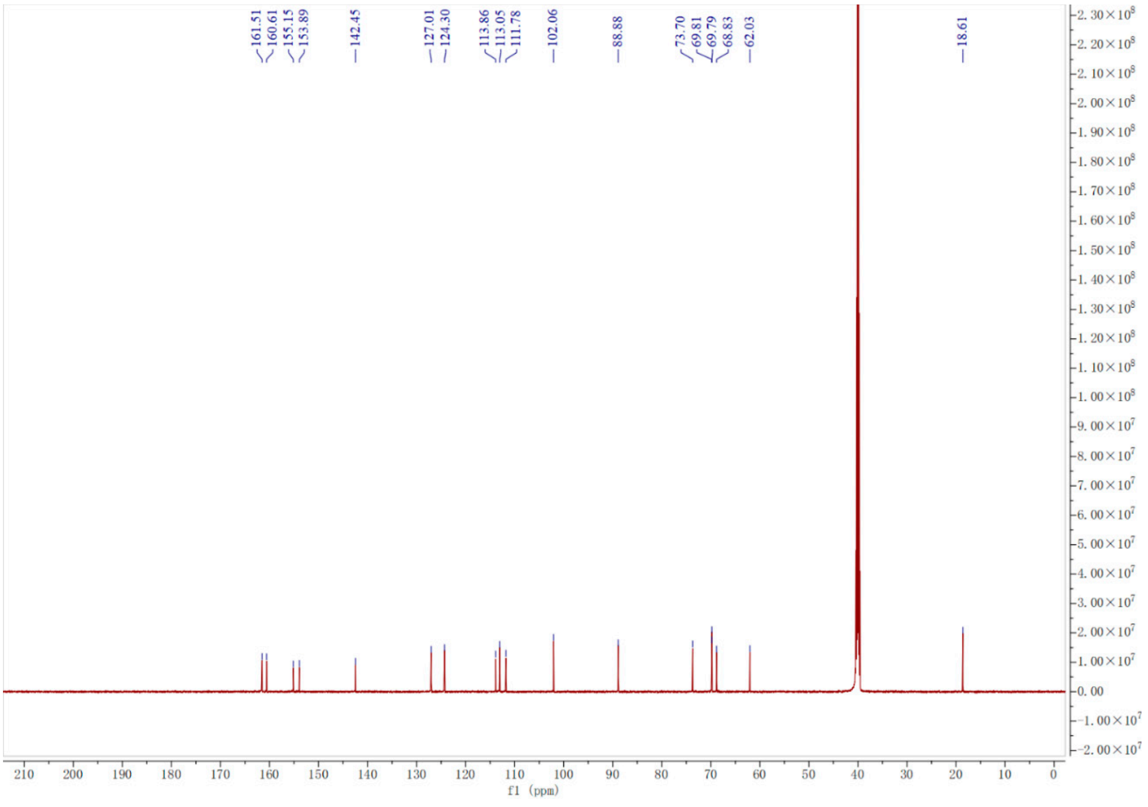

Figures S29. The ^{1}H-NMR spectrum of 7- [(1-β-L-rhamnosyl-1H-1,2,3-triazol-4-yl) methoxy]-4-methyl-2H-chromen-2-one

(10o)

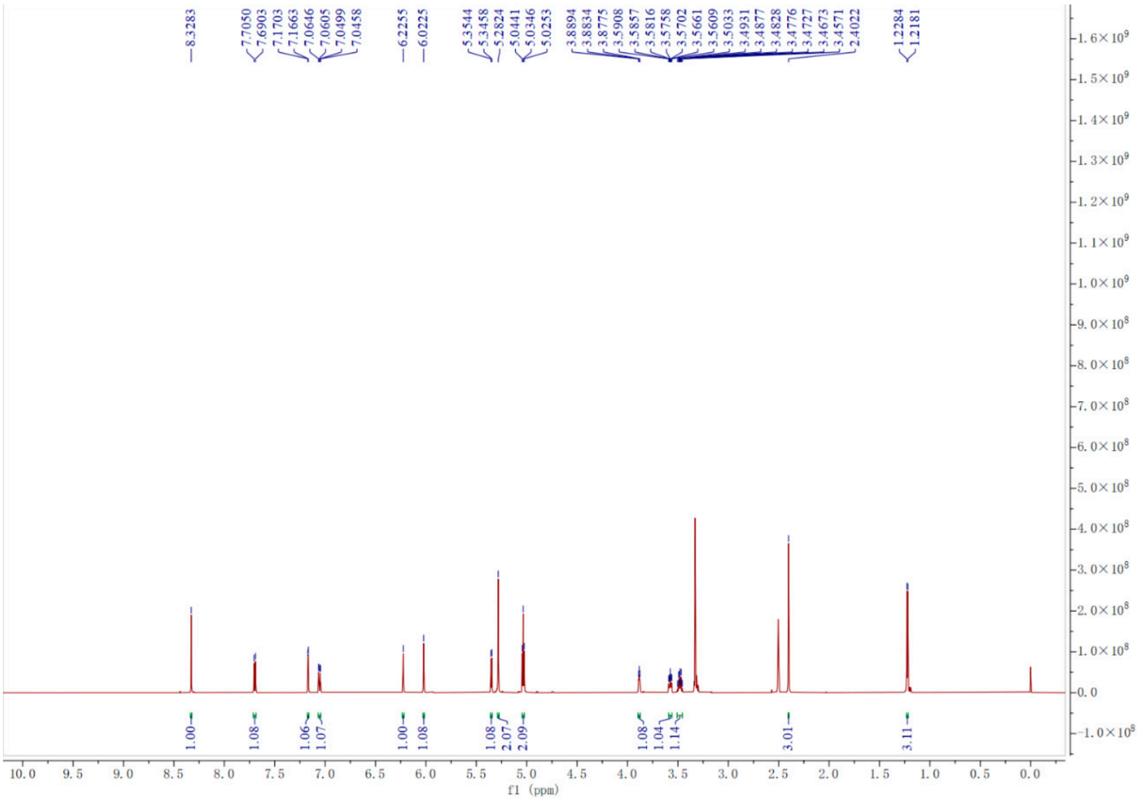

Figures S30. The ^{13}C-NMR spectrum of 7- [(1-β-L-rhamnosyl-1H-1,2,3-triazol-4-yl) methoxy]-4-methyl-2H-chromen-2-one

(10o)

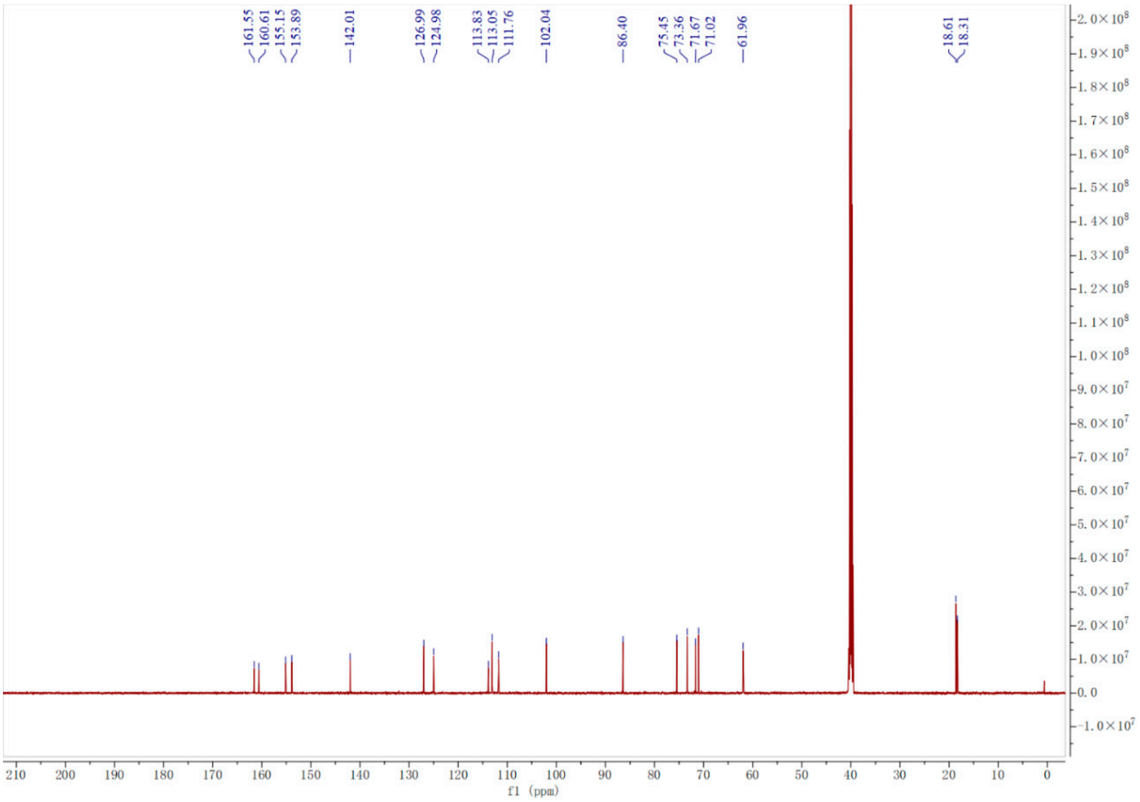

Figures S31. The ¹H-NMR spectrum of 7- [(1-β-D-ribofuranosyl-1H-1,2,3-triazol-4-yl) methoxy]-4-methyl-2H-chromen-2-one

(10p)

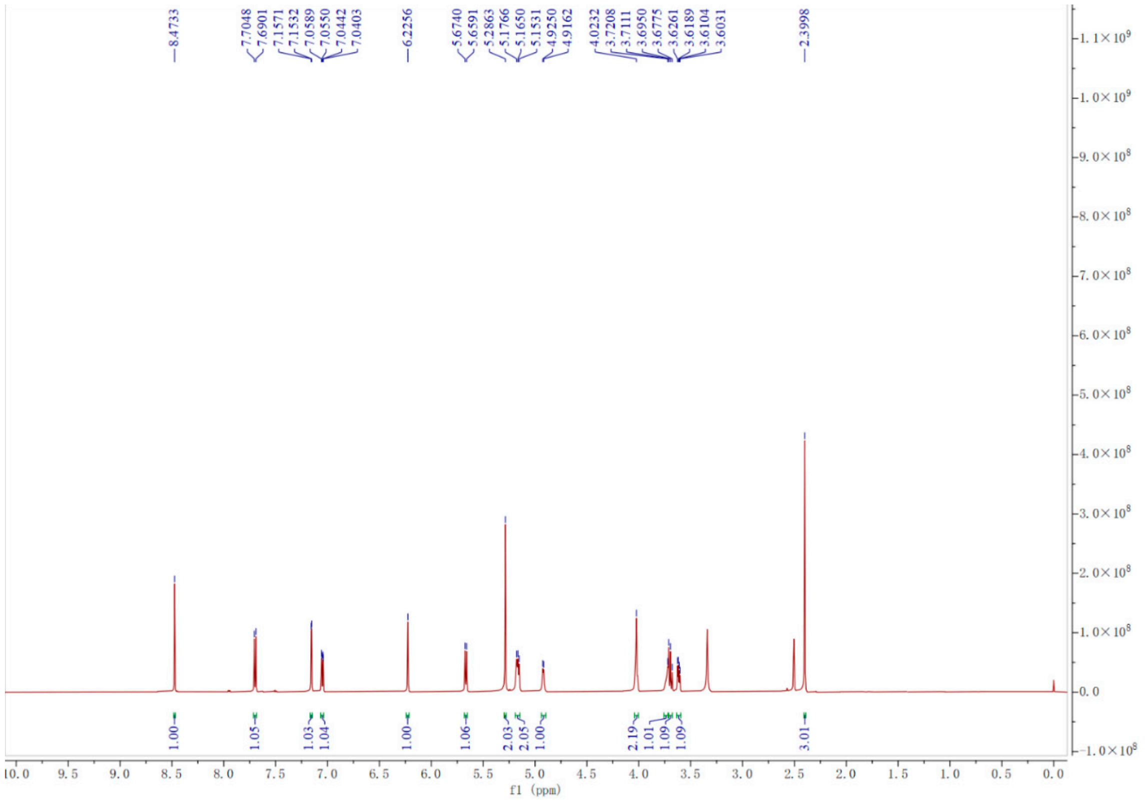

Figures S32. The ¹³C-NMR spectrum of 7- [(1-β-D-ribofuranosyl-1H-1,2,3-triazol-4-yl) methoxy]-4-methyl-2H-chromen-2-one

(10p)

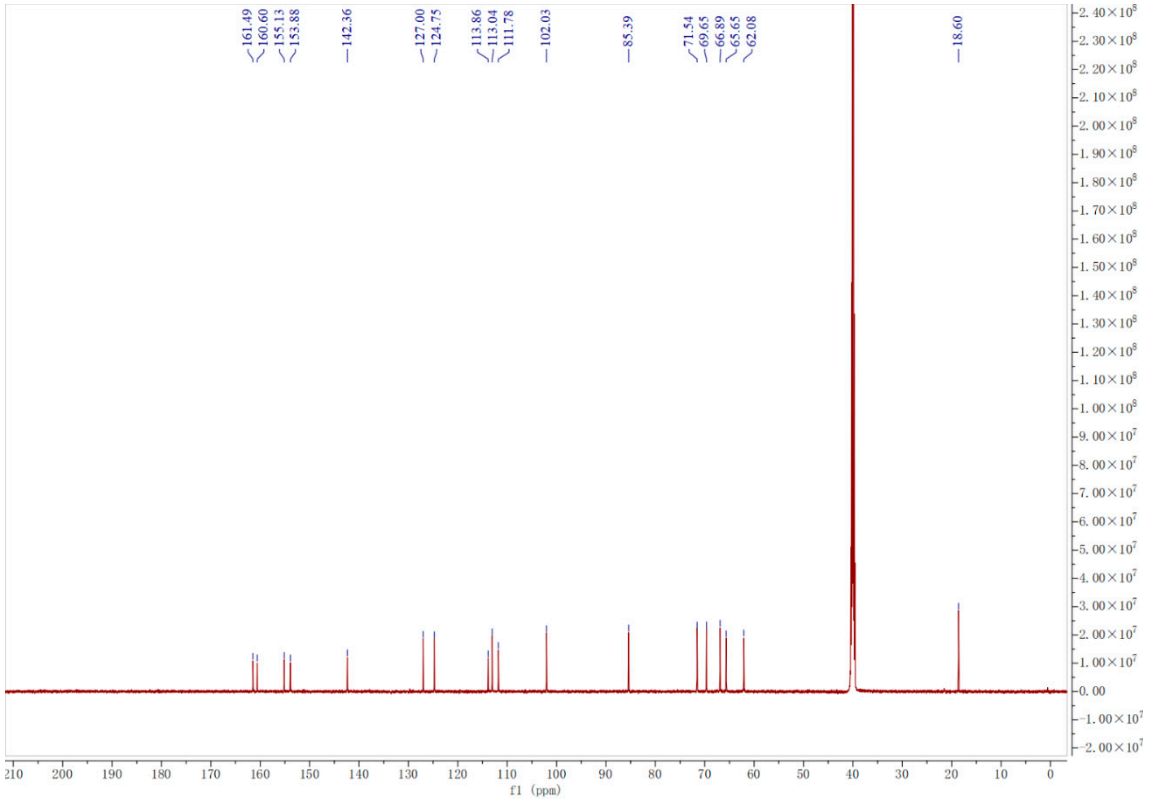

Supplement: Supplementary file 1 [file molecules-27-05464-s001.zip › molecules-1863784-supplementary.pdf]
